# Ectopic thyroid papillary carcinoma of nasopharynx associated with adenoid hypertrophy: an unusual presentation

**DOI:** 10.1186/1746-160X-10-40

**Published:** 2014-09-20

**Authors:** Linli Tian, Yufei Jiao, Ming Liu, Minghua Li, Hongchao Yao

**Affiliations:** Department of Otolaryngology, Head and Neck Surgery, The Second Affiliated Hospital, Harbin Medical University, Harbin, China; Department of Pathology, The Second Affiliated Hospital, Harbin Medical University, Harbin, China; Department of Otorhinolaryngology, Head and Neck Surgery, Second Affiliated Hospital, Harbin Medical University, Harbin, 150081 China

**Keywords:** Ectopic thyroid papillary carcinoma, Nasopharynx, Adenoid hypertrophy, Nasal obstruction

## Abstract

Ectopic thyroid tissue of nasopharynx is an uncommon phenomenon and papillary thyroid carcinoma arising from the tissue is extremely rare. The authors report a rare case of 16-year-old girl with papillary thyroid carcinoma of nasopharynx. Clinicians were ever confused by adenoid hypertrophy and solved the diagnostic dilemma by adequate examinations. In the case, we mainly emphasize that surgeons should be aware of and actively consider such a possibility of ectopic papillary thyroid carcinoma of nasopharynx in children and adolescents with long-term nasal obstruction, even if thyroid carcinoma is a rare tumor.

## Introduction

Ectopic thyroid tissue is rare, which the reported incidence is 1 in every 100,000 to 300,000 in the general population [1, 2]. A thyroid gland can reside anywhere along thyroid embryologic path from the foramen cecum to its normal site such as lingua, thyroglossal duct and laryngotrachea [3]. The abnormal migration of the thyroid is known as ectopic thyroid [4]. Ectopic thyroid tissue, especially ectopic papillary thyroid carcinoma (PTC) of nasopharynx, is extremely rare, and may cause diagnostic and therapic dilemma for clinicians just as our case. To our knowledge, very few reports of ectopic nasopharyngeal thyroid cancer with a normal eutopic thyroid gland have been published to date [5, 6]. Herein we present an uncommon case of ectopic PTC of nasopharynx associated with adenoidal hypertrophy and share our experience of the successful management about such a rare case.

## Case report

**Figure 1:**
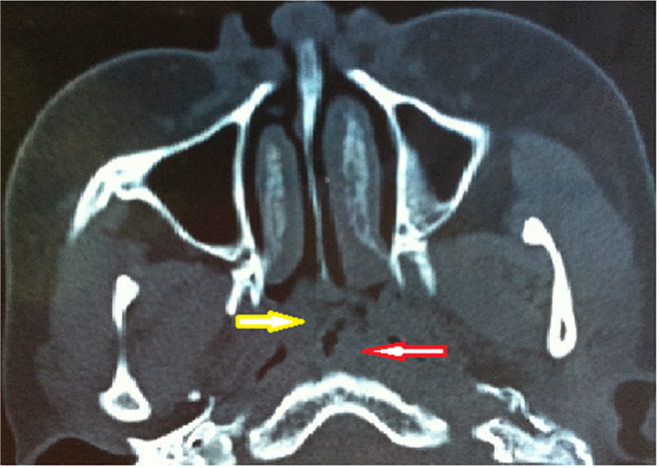
**Preoperative CT findings of the patient.** Overlapping image of ectopic PTC (yellow arrow) and hypertrophic adenoid (red arrow) in nasopharynx.

**Figure 2:**
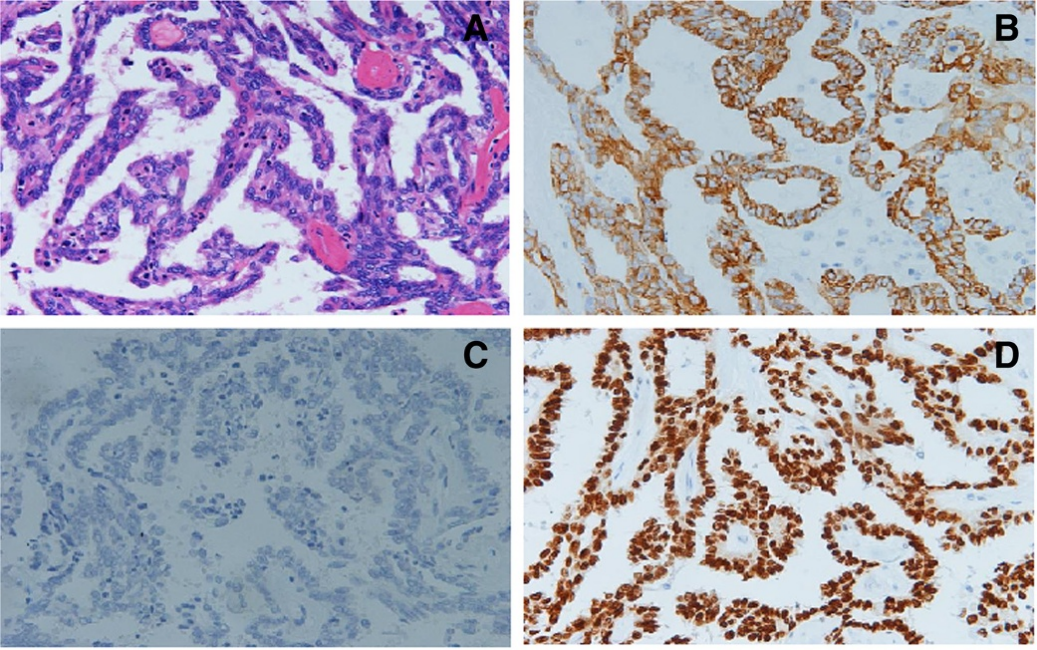
**Histopathological examination of thyroid showing papillary carcinoma. A**: H & E stain showed moderately pleomorphic malignant oval to rounded epithelial cells. **B**, **C**, **D**: Immunohistochemical analysis revealed positive CK **(B)**, TG **(C)** and TTF-1 **(D)** as markers of PTC. Magnification: ×200.

**Figure 3:**
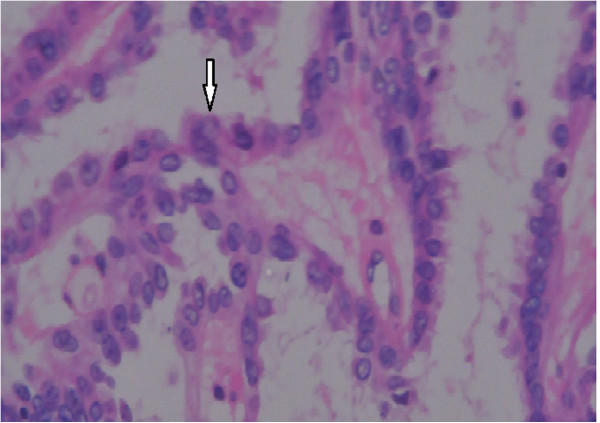
**H & E stain showed nuclear features such as ground glass nuclei and nuclear grooves (arrow).** Magnification: ×400.

A 16-year-old girl presented to the Department of ENT and Head and Neck Surgery at the Second Affiliated Hospital of Harbin Medical University with a history of persistent nasal obstruction of 7 years duration. The girl was diagnosed with adenoid hypertrophy 5 years ago by simple CT scan examination (unavailable). At that time, the patient’s family refused any further examinations and treatment for fear of surgery. By this time, clinical examination revealed no pyrexia, heart rate 90 bpm and normal life signs. Physical examination revealed a nasopharyngeal mass blocking the majority of postnaris. Nasal endoscopic examination found oval mass with pedicle located in the nasopharyngeal posterior wall across hypertrophic adenoid. The tumor was smooth, enveloped with a clear demarcation. A CT scan showed a solid cystic mass located in the nasopharynx (Figure 1). The thyroid gland was normal and no cervical lymphadenopathy was noted. Following biopsy pathology, papillary thyroid carcinoma was diagnosed (Figures 2 and 3). Tumor resection was performed through FESS under general anesthesia 3 days later. Postoperative pathological examination further confirmed papillary thyroid carcinoma in the nasopharyngeal mass with histopathological features of pleomorphic malignant oval to rounded epithelial cells (Figure 2A) and ground glass nuclei and nuclear grooves of cells (Figure 3). Immunohistochemical analysis revealed positive cytokeratin (CK), thyroglobulin (TG) and thyroid transcription factor-1 (TTF-1) as diagnostic PTC markers (Figure 2B,C,D). The postoperative period was uneventful and the patient was discharged from the hospital 5 days later. The patient could not be treated with thyroidectomy and radioactive iodine therapy because relatives of patient refused the recommendation. Upon follow-up at 6 months, the patient remains asymptomatic.

## Discussion

Thyroid carcinoma is a relatively rare pediatric pathology, comprising around 3% of children and adolescents tumors [7]. Papillary thyroid carcinoma, a kind of differentiated thyroid cancer, is the most common neoplasm in the thyroid gland and accounts for about 80% of all thyroid cancers [8]. Ectopic papillary thyroid carcinoma has been found in some places such as lingua [3], mediastinum [9] and thyroglossal duct [10]. Ectopic PTC of nasopharynx is extremely scarce especially with normal eutopic thyroid gland and no lymph node involvement just as our case.

The girl of our case had long-term nasal obstruction and was diagnosed as adenoid hypertrophy before 5 years by means of simple CT scan examination. Because of increasing nasal obstruction, the patient was checked by further examinations including CT scan and endoscopy and an oval neoplasm with intact capsule was found to be located on nasopharynx near hypertrophic adenoid. This case suggested that nasal obstruction of children and adolescent could be caused by not only lymphoid tissue hyperplasia and common neoplasms but also unusual tumors. Then, biopsy showed histological characteristics of papillarity, ground glass nuclei and nuclear grooves of cells, suggesting this was a thyroid-like tumor of malignant origin. Additional examination of immunohistochemistry revealed that the positive expressions of TTF-1, CK and TG and negative for P63. TTF-1 is currently used in routine surgical pathology as an immunohischemical marker of primary carcinomas arising in thyroid and lung organs. It has also been reported to be expressed in other tumors such as thymoma, ovarian, endocervical and endometrial neoplasms [11, 12]. According to the histological characteristics and immunohistochemical features, we made a final diagnosis of papillary thyroid carcinoma on nasopharynx. Other thyroid-like tumors such as low-grade papillary adenocarcinoma of the nasopharynx (LGPACNP) also exhibit similar features [13, 14]. However, the fact that the positive expression of TG has hitherto not been mentioned in previous reports of LGPACNP could exclude this diagnostic possibility [15]. In addition, normal thyroid gland in its pretracheal position and no cervical lymphadenopathy gave us evidence to support our diagnosis of ectopic PTC rather than metastasis of thyroid carcinoma [16].

Treatment outcomes of PTC are very favorable and have an excellent prognosis with 10-year survival rates of more than 90% [17, 18]. The optional treatment methods have surgery, radiotherapy, radioactive iodine therapy, and chemotherapy according to the current management guidelines for patients with differentiated thyroid cancer issued by the American Thyroid Association [19]. Adequate surgical treatment followed by postoperative radioactive iodine therapy can offer very promising results for ectopic PTC.

## Conclusion

In conclusion, even if thyroid carcinoma is a rare tumor in children and adolescents, surgeons should be aware of and actively consider such a possibility of ectopic papillary thyroid carcinoma of nasopharynx in patients with nasal obstruction. Since the clinical signs and symptoms are most often vague mimicking other nasopharyngeal diseases, ectopic thyroid carcinoma should be considered in the differential diagnosis even in the presence of a normal thyroid gland. Imageological examinations such as CT scan and biopsy are usually necessary for diagnosis and treatment. The present case report shows that there is always a chance to encounter exceedingly rare disease and adequate examinations of pre-operation are necessary for appropriate diagnosis and treatment.

## Consent

Written informed consent was obtained from the patient’s next of kin for publication of this case report and any accompanying images. A copy of the written consent is available for review by the Editor-in-Chief of this journal. The review board of Harbin Medical University approved publication of this case report.
